# Analysis on the intention and influencing factors of free influenza vaccination among the elderly people aged 70 and above in Hangzhou in 2022

**DOI:** 10.3389/fpubh.2022.1052500

**Published:** 2023-01-06

**Authors:** Xinren Che, Yan Liu, Wenwen Gu, Fangying Wang, Jun Wang, Wei Jiang, Jian Du, Xiaoping Zhang, Yuyang Xu, Xuechao Zhang, Jing Wang

**Affiliations:** ^1^Department of Expanded Program on Immunization, Hangzhou Center for Disease Control and Prevention, Hangzhou, Zhejiang, China; ^2^School of Public Health, Hangzhou Normal University, Hangzhou, Zhejiang, China

**Keywords:** attitudes, influencing factors, influenza vaccines, elderly people, Hangzhou, China

## Abstract

**Background:**

Although influenza vaccination is recommended for people aged 70 and above in Hangzhou, and the vaccine is provided free of charge, the elderly influenza vaccination rate is still low. The purpose of this study was to understand the barriers and motivations of older people in deciding to receive free influenza vaccine through questionnaires.

**Methods:**

The method of stratified random sampling was adopted to take samples. A questionnaire survey was conducted among the elderly aged 70 years and above by face-to-face interview or telephone interview.

**Results:**

A total of 11,663 elderly people aged 70–100 years were successfully and effectively interviewed. 85.98% of the respondent were willing to get the influenza shot, 8.91% were unwilling to get the influenza shot, and 5.11% were on vaccine hesitancy. The people of age of 70–79 years old (hesitancy: *OR*_70~79_ = 0.668, 95%*CI*: 0.571 0.782, Unwilling: *OR*_70 − 79_ = 0.755, 95%*CI*: 0.622 0.916), primary school degree or below (hesitancy: *OR*_Secondary school degree or above_ = 1.467, 95%*CI*: 1.249 1.724, Unwilling: *OR*_Secondary school degree or above_ = 1.255, 95%*CI*: 1.028 1.535), remote areas (hesitancy: *OR*_near central urban area_ = 2.111, 95%*CI*: 1.604 2.778, *OR*_central urban area_ = 2.957, 95%*CI*: 2.255 3.877, Unwilling: *OR*_near central urban area_ = 1.687, 95%*CI*: 1.230 2.313. *OR*_centralurbanarea_ = 2.218, 95%*CI*: 1.626 3.027), and convenient for movement (hesitancy: *OR*_yes_ = 0.494, 95%*CI*: 0.420 0.580, Unwilling: *OR*_yes_ = 0.585, 95%*CI*: 0.480 0.713), understanding of the free vaccine policy (hesitancy: *OR*_understand_ = 0.204, 95%*CI*: 0.171 0.245, Unwilling: *OR*_understand_ = 0.164, 95%*CI*: 0.128 0.210), influenza knowledge level≥ 13 points (hesitancy: *OR*_≥13*points*_ = 0.628, 95%*CI*: 0.533 0.739, Unwilling: *OR*_≥13*points*_ = 0.538, 95%*CI*: 0.437 0.662), influenza vaccine knowledge level≥ 12 points (hesitancy: *OR*_≥12*points*_ = 0.422, 95%*CI*: 0.350 0.508, Unwilling: *OR*_≥12*points*_ = 0.370, 95%*CI*: 0.290 0.472), and social trust level ≥ 12 points (hesitancy: *OR*_≥12*points*_ = 0.134, 95%*CI*: 0.112 0.160, Unwilling: *OR*_≥12*points*_ = 0.220, 95%*CI*: 0.180 0.269) are more willing to receive free influenza vaccine.

**Conclusion:**

The proportion of elderly people aged 70 and above who are willing to receive free influenza vaccine is high in Hangzhou. But the level of knowledge about influenza, vaccine and trust in society is low. The government should continue to improve the elderly's awareness and trust in society through medical staff, family members, television and radio media, and guide the elderly to actively vaccinate against influenza. Effective publicity should be carried out through the above channels to guide the elderly to actively vaccinate against influenza.

## 1. Introduction

Influenza is an acute respiratory infectious disease caused by the influenza virus. Influenza has a high incidence in the population and has caused many outbreaks around the world. Influenza patients have mild upper respiratory tract catarrhal symptoms, but severe systemic symptoms such as high fever, headache, and fatigue (1). Elderly people are more susceptible to influenza because of their weakened immune function and prevalence of chronic diseases (2). Elderly influenza patients often suffer from severe pulmonary infection, which aggravates organ disease and is one of the main causes of death (3).

Studies have shown that population vaccination against influenza can reduce outbreaks of an influenza-like illness (4) and reduce hospital readmissions in older adults with cardiovascular or respiratory diseases (5). Antibodies produced after an influenza vaccination in frail older adults may disappear (6), but the vaccine can still reduce hospitalizations and deaths in these susceptible populations (7–9). Studies have also shown an inverse association between influenza vaccination coverage and SARS-CoV-2 seroprevalence, prevalence of hospitalized patients, prevalence of patients admitted to intensive care units, and deaths attributed to COVID-19 (10). Seasonal influenza vaccinations may have some influence on the incidence and severity of COVID-19 outbreaks (11). With or without additional protective benefits, an influenza vaccination is cost-effective compared with no vaccination (12). Influenza vaccinations for adults 60 years of age or older are highly cost-effective (13). At present, influenza vaccinations are still one of the most effective measures to prevent the occurrence and epidemic of influenza (14). Both the WHO (15) and National Immunization Advisory Committee Technical Working Group (16) recommend influenza vaccinations and that the annual influenza vaccine should be allocated to the elderly and other high-risk groups as priority before the rest of the population.

The average annual vaccination rate for influenza vaccine in China is only 2–3%. In most areas, influenza vaccinations require paying for themselves. Only a few provinces and cities, such as Beijing, Shanghai, Karamay in Xinjiang Province, Shenzhen in Guangdong province and Xinxiang in Henan province, have implemented free influenza vaccinations for the elderly or children (17).some scholars proposed that there should be financing strategies for influenza vaccinations for the elderly (18), including the individual-central-local mechanism (strategy 1), central-local mechanism (strategy 2) and local payment mechanism (strategy 3). Strategy 3 is the current elderly influenza vaccine payment strategy implemented in Hangzhou, Zhejiang Province, which is paid by the local government. Since 2020, Hangzhou has started to provide free influenza vaccines to individuals with a Zhejiang household registration who are 70 years old and above. About 250,000 people are vaccinated each year. In addition, about 50,000 elderly people aged 60 and above receive influenza vaccines at their own expense each year in Hangzhou, and the influenza vaccine coverage rate for this age group is about 15%. In Hangzhou and other parts of China (19), influenza vaccination rates among the elderly are still low. Studies involving the willingness of the elderly to be vaccinated, have shown that multi-component interventions, such as reminders and persuasion to vaccinate at a community-level, can help increase the vaccination rate and reduce the incidence of influenza in the elderly (20). This paper analyzes the reasons of influencing the free influenza vaccination for the elderly people aged 70 and above in Hangzhou. The aim is to prevent influenza through improve the influenza vaccination rate of the elderly population in Hangzhou.

## 2. Methods

### 2.1. Basic information and definitions

According to the distance from the central city of Hangzhou, it can be divided into central urban area, near central urban area and remote area. There are 14 districts or counties within Hangzhou, of which Xihufengjingmingsheng has a small population aged 70 and above and did not participate in the survey, 5 are classified as central urban areas (Shangcheng, Gongshu, Xihu, Binjiang and Qiantang), 5 are classified as near central urban area (Xiaoshan, Yuhang, Linping, Fuyang and Linan), and the rest are classified as remote areas (Tonglu, Jiande, Chunan).

Hesitation refers to vaccine hesitation, which refers to the delay or refusal of vaccination due to the lack of awareness of vaccine safety, effectiveness, and disease prevention.

Adverse event following immunization (AEFI) refers to the reaction during or after vaccination that may cause damage to tissues, organs, or functions of the recipients that are suspected to be related to vaccination.

### 2.2. Sampling method

The method of stratified random sampling was adopted to take samples from 13 districts and counties in equal proportion. The function n = (*Z*_1−α/2_/δ)^2^p(1-p) was used to calculate the overall sample size. the sample population aged 80 years and above was not <25% of the total sample population (at present, the elderly over 80 years old in Hangzhou account for 25% of the total number of elderly people aged 70 and above.). The number of people aged 70 and above was obtained from the 2021 population data from the “Chinese Information System for Disease Control and Prevention”. We estimated a sample size of 9,604 participants, based on α = 1.96, percentage willing to get influenza vaccine (p) = 50%, maximum permissible error (δ) = 0.01p. The total number of elderly people aged 70 and above was 1,172,655. According to the formula *k* = n/N (k is the sampling proportion, *N* is the number of individuals in the population, n is the sample size), the sampling proportion is 0.819%.

### 2.3. Survey methods

A self-designed questionnaire was used in the survey. The 13 district and county centers for Disease Control and prevention in Hangzhou conducted face-to-face surveys or telephone surveys through community workers or medical staff from the community healthcare center. After the survey, the written responses were entered into online web pages for data collection. The survey was conducted on a voluntary basis. After informed consent, the respondents answered the questions and the investigators filled out the questionnaire.

### 2.4. Survey questionnaire

We surveyed selected older adults with a standardized, anonymous questionnaire. The questionnaire topics included sociodemographic characteristics, previous health status, knowledge of influenza and vaccines, intention of influenza vaccination in 2022, and social trust level. The responses to the questions regarding “Influenza knowledge level”, “Influenza vaccine knowledge level”, and “social trust level” were assigned with fixed scoring principles (Table 1).

**Table 1:** Fixed scoring principles.

	**Topic/options**	**Yes**	**Uncertain**	**No**
Influenza knowledge level	1) There is a difference between influenza and a cold	3	2	1
	2) Influenza in the elderly can cause complications	3	2	1
	3) Influenza is a common infectious disease	3	2	1
	4) Influenza vaccine in older adults reduces pneumonia risk	3	2	1
	5) Influenza is less contagious	1	2	3
Influenza vaccine knowledge level	1) Have you heard of the Influenza vaccine?	3	2	1
	2) Is the Influenza vaccine available every year?	3	2	1
	3) Getting the Influenza vaccine protects others (family members) from contracting Influenza	3	2	1
	4) Influenza vaccines are not safe	1	2	3
	5) Influenza vaccines are prone to serious side effects	1	2	3
Social trust level	1) Do you trust government-organized free Influenza vaccination programs?	3	2	1
	2) Will you be participating in the free Influenza vaccination program because of a doctor's recommendation?	3	2	1
	3) Are you participating in the free Influenza vaccination program because of a referral from a family member or friend?	3	2	1
	4) Will you participate in the free Influenza vaccination program because of the participation of those around you?	3	2	1

### 2.5. Statistical analysis technique

SPSS 21.0 (IBM Corporation, New York, USA) software was used for data analysis. Counting data are described by frequency and composition ratio. Chi-square test, univariate and multivariate Logistic regression analysis were used for data analysis, with test level α = 0.05.

### 2.6. Ethical considerations

This study was approved by the Hangzhou CDC institutional review board. Both the processes involving data collection and the final storage of data are secure. The names of the interviewees have been anonymous in all the publicly available data.

## 3. Result

### 3.1. Demographics

A total of 11,740 questionnaires were sent out and 11,663 were collected. The response rate and validity of the questionnaire were 99.34 and 100%, respectively. 11,663 people were surveyed, including 5,723 males and 5,940 females, with a sex ratio of 1:1.04. Due to the use of face-to-face surveys or telephone surveys by community workers or medical staff from a community healthcare center, and the strict control over the quality of each questionnaire, all 11,663 questionnaires were valid surveys. The average age of the respondents was 76.93±5.58 years old, of which 72.7% were 70–79 years old, and 27.3% were over 80 years old. The minimum age of the respondents was 70 years old, and the maximum age was 100 years old. The educational level of the respondents was mostly primary school or below, accounting for 67.0%. 71.9% of the respondents had a history of chronic diseases. The educational level of the elderly over 70 years old is relatively low, with only 33% having secondary school degree or above. 72% of the elderly over 70 years old suffer from cardiovascular and cerebrovascular diseases, tumor, chronic respiratory disease, diabetes and other chronic diseases (Table 2).

**Table 2:** Survey on influenza vaccination intention among the elderly over 70 years old in Hangzhou.

**Demographic characteristics**	**No. of survey**	**Proportion (%)**
**Gender**		
Male	5,723	49.1
Female	5,940	50.9
**Age (year)** ^a^		
70–79	8,480	72.7
≥80	3,183	27.3
**Region**		
Shangcheng	1,591	13.6
Gongshu	1,636	14.0
Xihu	1,047	9.0
Binjiang	236	2.0
Xiaoshan	1,094	9.4
Yuhang	537	4.6
Linping	892	7.6
Fuyang	1,149	9.9
Linan	812	7.0
Tonglu	717	6.1
Jiande	861	7.4
Chunan	642	5.5
Qiantang	449	3.8
**Degree of education**		
Primary school degree or below	7,809	67.0
Secondary school	2,386	20.5
High school	901	7.7
Associate degree	313	2.7
University and above	254	2.2
**Living status (Live with family or friends)**		
Live alone	2,779	23.8
2 people live together	4,022	34.5
3 people live together	1,429	12.3
4 people live together	1,303	11.2
≥5 people live together	2,130	18.3
**Chronic medical conditions**		
Cardiovascular and cerebrovascular diseases	5,789	42.7
Tumor	362	2.7
Chronic respiratory disease	952	7.0
Diabetes	1,673	12.3
Other chronic diseases	1,974	7.2
Nothing	3,274	24.1
Unaware	533	3.9

a The age is 76.93 ± 5.58, the maximum value is 100, the minimum value is 70.

### 3.2. Univariate analysis of vaccination willingness

Among all the respondents, 10,028 (85.98%) were willing to receive a free influenza vaccine; 1,039 (8.91%) were reluctant to receive the free influenza vaccine, and 596 (5.11%) were hesitant. The willingness rate of influenza vaccination varied by age, region, degree of education, chronic medical conditions, convenience, understanding of the free vaccine policy, influenza knowledge level, influenza vaccine knowledge level, and social trust level. The differences were statistically significant (*P* < 0.05). Among them, people in urban areas, near central urban area and remote areas have different vaccination intentions, 80.76% (4005/4959), 87.78% (4484/3936) and 94.01% (2220/2087), respectively (Table 3).

**Table 3:** Univariate analysis of the willingness of the elderly aged 70 years and above to receive free influenza vaccination in Hangzhou.

**Demographic characteristics**	**No. of survey**	**Intention to vaccinate (yes/no/ hesitation)**			**χ^2^-value**	***P*-value**
		**Yes**	**No**	**Hesitation**		
**Gender**					10.455	0.005
Male	5,723	4,972	494	257		
Female	5,940	5,056	545	339		
**Age (year)**					70.445	<0.001
70–79	8,480	7,430	657	393		
≥80	3,183	2,598	382	203		
**Region**					244.535	<0.001
Central urban area	4,959	4,005	617	337		
Near central urban area	4,484	3,936	346	202		
Remote area	2,220	2,087	76	57		
**Degree of education**					41.265	<0.001
Primary school degree or below	7,809	6,822	609	378		
Secondary school degree or above	3,854	3,206	430	218		
**Chronic medical conditions**					44.601	<0.001
Yes	5,775	4,842	582	351		
No	5,888	5,186	457	245		
**Whether the action convenience**					276.515	<0.001
Yes	9,385	8,316	671	398		
No	2,278	1,712	368	198		
**Whether understanding of the free vaccine policy**					1,465.813	<0.001
Understand	6,930	6,663	182	85		
Do not understand	4,733	3,365	857	511		
**Influenza knowledge level**					535.979	<0.001
≥13	6,451	5,977	318	156		
<13	5,212	4,051	721	440		
**Influenza vaccine knowledge level**					933.481	<0.001
≥12	6,064	5,786	185	93		
<12	5,599	4,242	854	503		
**Social trust level**					1,351.470	<0.001
≥12	7,161	6,826	185	150		
<12	4,502	3,202	854	446		

### 3.3. Multivariate analysis of vaccination willingness

Using the willingness to receive an influenza vaccination as the dependent variable, a multivariate Logistic regression analysis was performed on the variables where P <0.05. The results show that whether it is a comparative analysis of those who are willing to be vaccinated and those who are hesitant to be vaccinated, or a comparative analysis of those who are willing to be vaccinated and those who are unwilling to vaccinate, it is in line with the age of 70–79 years old, primary school degree or below, remote areas, and convenient for movement, understanding of the free vaccine policy, influenza knowledge level ≥13 points, influenza vaccine knowledge level ≥12 points, and social trust level ≥12 points are more willing to receive free influenza vaccine (Table 4).

**Table 4:** Multivariate analysis of willingness to receive free influenza vaccine among the elderly aged 70 and above in Hangzhou.

**Demographic characteristics**	**Reference**	**β-value**	**S.E**.	**Wals**	**OR**	**95%CI**	***P*-value**
**Vaccination hesitancy**						
**Gender**	Female						
Male		−0.036	0.075	0.235	0.964	0.832 1.117	0.627
**Age (year)**	≥80						
70–79		−0.403	0.080	25.254	0.668	0.571 0.782	<0.001
**Degree of education**	Primary school degree or below						
Secondary school degree or above		0.383	0.082	21.696	1.467	1.249 1.724	<0.001
**Region**	Remote area						
Near central urban area		0.747	0.140	28.413	2.111	1.604 2.778	<0.001
Central urban area		1.084	0.138	61.500	2.957	2.255 3.877	<0.001
**Chronic medical conditions**	No						
Yes		0.010	0.076	0.017	1.010	0.871 1.172	0.871
**Convenience**	No						
Yes		−0.706	0.083	73.111	0.494	0.420 0.580	<0.001
**Understanding of the free vaccine policy**	Do not understand						
Understand		−1.587	0.092	297.012	0.204	0.171 0.245	<0.001
**Influenza knowledge level**	<13						
≥13		−0.466	0.083	31.254	0.628	0.533 0.739	<0.001
**Influenza vaccine knowledge level**	<12						
≥12		−0.863	0.095	92.948	0.422	0.350 0.508	<0.001
**Social trust level**	<12?						
≥12		−2.010	0.089	505.557	0.134	0.112 0.160	<0.001
**Unwilling to vaccinate**						
**Gender**	Female						
Male		−0.195	0.092	4.466	0.823	0.687 0.986	0.035
**Age (year)**	≥80						
70–79		−0.281	0.099	8.113	0.755	0.622 0.916	0.004
**Degree of education**	Primary school degree or below						
Secondary school degree or above		0.227	0.102	4.982	1.255	1.028 1.535	0.026
**Region**	Remote area						
Near central urban area		0.523	0.161	10.537	1.687	1.230 2.313	0.001
Central urban area		0.797	0.157	25.256	2.218	1.626 3.027	<0.001
**Chronic medical conditions**	No						
Yes		0.166	0.093	3.155	1.180	0.983 1.417	0.076
**Convenience**	No						
Yes		−0.537	0.101	28.315	0.585	0.480 0.713	<0.001
**Understanding of the free vaccine policy**	Do not understand						
Understand		−0.809	0.126	207.008	0.164	0.128 0.210	<0.001
**Influenza knowledge level**	<13						
≥13		−0.620	0.106	34.027	0.538	0.437 0.662	<0.001
**Influenza vaccine knowledge level**	<12						
≥12		−0.995	0.125	63.535	0.370	0.290 0.472	<0.001
**Social trust level**	<12						
≥12		−1.513	0.103	217.440	0.220	0.180 0.269	<0.001

### 3.4. Reasons for not getting the free influenza vaccine

A questionnaire survey was conducted among 1,039 respondents who were not willing to receive an influenza vaccine. It was found that the main reasons for not being willing to receive an influenza vaccine were fear of side effects after vaccination, inconvenience to go for a vaccination, uncommon influenza and low risk of influenza infection, accounting for 22, 15.1, 12.6, and 12.0%, respectively (Table 5).

**Table 5:** Reasons why the elderly aged 70 years and above in Hangzhou are not willing to receive free influenza vaccines.

**Reasons**	**Cumulative number**	**Proportion (%)**
Influenza is uncommon	301	12.6
The risk of influenza is small	287	12.0
Even if I get the influenza, the symptoms won't be serious	176	7.4
I don't have to get the influenza shot if other people get the influenza shot	78	3.3
The influenza vaccine is not safe	136	5.7
The influenza vaccine is not effective	112	4.7
Worry about AEFI after inoculation	525	22.0
It is not convenient to travel to vaccinate	360	15.1
Vaccine brands cannot be selected	33	1.4
Distrust the activities of government organizations	10	0.4
I have contraindications for influenza vaccination	176	7.4
Others	191	8.0

### 3.5. Access to health information

A questionnaire survey on the ways of obtaining health information among 11,663 elderly people aged 70 years and above showed that medical institutions and medical staff, family, friends, television and radio were the main ways of obtaining health information, accounting for 27.3, 26.7, and 22.1%, respectively (Table 6).

**Table 6:** Statistical table of ways for the elderly aged 70 years and above to obtain health information in Hangzhou.

**Approach**	**Cumulative number**	**Proportion (%)**
Newspapers or books	2,533	9.7
Television or Radio	5,784	22.1
Computer network platform	1,260	4.8
Mobile apps such as wechat, Weibo, et al.	1,959	7.5
Medical institutions and medical staff	7,127	27.3
Family or friends	6,975	26.7
Others	505	1.9

## 4. Discussion

The study shows that a high proportion of people over 70 years old in Hangzhou are willing to get the free influenza vaccine. The proportion of elderly people willing to take an influenza vaccine in Hangzhou is relatively high, which is similar to the study results from the Jiaojiang District of Taizhou City (21) and Chongqing City (22), revealing that elderly people are subjectively willing to take an influenza vaccine. The elderly population with a secondary school degree or above had a lower willingness or more hesitation in taking an influenza vaccination, which was contrary to the results of Peng-Jun Lu (23), Qiushuang Li et al. (24) This may be related to the fact that people with higher education are more likely to form their own personal opinions and have vaccine hesitation after fully understanding the benefits of vaccination and possible AEFI. News media reports of a small number of severe AEFIs have heightened concerns about vaccination among the highly educated. Although the education level of the elderly over 70 years old in Hangzhou is generally not high, the phenomenon of refusing influenza vaccinations among the well-educated elderly population in the Hangzhou area deserves our reflection and further research. The central urban area and near central urban area elderly population in the Hangzhou area had a lower willingness to vaccinate against influenza, contrary to the situation in Poland reported in the literature (25). Although the per capita income of central urban area residents in Hangzhou is similar to that in Poland (74,700 yuan/16,700 U.S. dollars), the willingness of central urban area residents in the two places to be vaccinated is completely opposite, which deserves further study.

Foreign studies have found that there is a strong correlation between free vaccine and vaccination intention (26, 27). In China, where the burden of seasonal influenza is high and vaccination rates are extremely low, the study suggests that most participants expect the government and/or health insurance to partially cover the costs (28). The policy of free influenza vaccination for the elderly aged 70 and above in Hangzhou has successfully improved the vaccination rate. This policy provides policy reference for influenza vaccination of the elderly in other regions of China.

Among all the reasons for unwillingness to choose an influenza vaccination, “Worry about AEFI after inoculation” (22%) had the highest proportion among all reasons for unwillingness to vaccinate, which was consistent with the results of the study in Shanghai, China (29). This may be because most people aged 70 and above suffer from chronic diseases, and AEFI can lead to recurrence or exacerbation of chronic diseases. The results of the Shanxi Province study in China showed that older people preferred influenza vaccines with a longer protection duration, followed by lower serious adverse events and higher vaccine effectiveness (30), which were also similar to the results of this study. Abroad, the Tunisian study found that the two main reasons for refusing vaccinations among older adults were fear of side effects (71.5%) and belief that vaccines were ineffective in avoiding influenza (33.9%) (31). In South Korea, a report of a death allegedly related to an Influenza vaccination in 2020 got a lot of media attention (32), followed by a sharp drop in influenza vaccination rates, from 80.5 to 83.3% in 2015–2019 to 73.6% in 2020–2021. These studies indicates that we should pay attention to people's concerns about AEFI. This may be an important reason why people refuse to be vaccinated against influenza. The degree to which people are concerned about AEFI directly affects people's willingness to vaccinate. The responses “influenza is uncommon ”and “the risk of influenza is small”accounted for 12.6 and 12.0%, respectively. This indicates that the elderly in Hangzhou have an incorrect understanding of influenza and lack of awareness of influenza vaccines, which affects the elderly population's choice of an influenza vaccine (33). It is consistent with the results of Aldiane Gomes de Macedo Bacurau and L M Teo (34), indicating that most older people do not consider influenza as a potentially serious disease and do not believe in the efficacy of an influenza vaccine. The misunderstanding of influenza knowledge and vaccine knowledge in the elderly plays an important role in the decline of influenza vaccinations, suggesting the need to increase the knowledge about influenza and influenza vaccines in the elderly. Providing necessary influenza counseling to those who cannot read, those who do not have a spouse/partner, and explaining the merits of vaccination to at-risk groups may help older adults choose an influenza vaccination.

This study found that medical institutions and medical staff, family, friends, television and radio are the main ways for the elderly to obtain health information. Education about influenza and influenza vaccines by doctors may promote influenza vaccine selection among older adults, thereby increasing influenza vaccination rates. These findings are consistent with those of Agnieszka Wozniak-Kosek (35), Natsuki Kajikawa (36), BirteBödeker (37), Shanxi Province, China (30). It is suggested that health care providers should be encouraged to focus on informing older adults about different aspects of influenza vaccines and influenza illness, as individual perceptions of the benefits of vaccination are critical in decision-making. Informing high-risk groups of influenza vaccination by medical institutions and health care workers also contributes to raising awareness of influenza and helping to increase vaccination rates (38). Recommendations from family and friends are also important factors influencing the elderly to get the influenza vaccine. Studies have shown that elderly people living alone have lower vaccination rates than those living with spouses or children (39), suggesting that health education should be carried out not only for the elderly, but also for their family members.

Similar to findings from other countries (40), this study also shows that improving health literacy through different communication approaches and ultimately informing the public about preventive measures such as vaccination remains one of the most significant public health challenges. Lack of public awareness, lack of adequate knowledge, lack of motivation, and lack of awareness of positive health behaviors including seasonal influenza vaccination remain the status quo of the current level of disease and vaccine awareness among older adults.

This study has some limitations. It shows that the elderly people have a high willingness to receive an influenza vaccination, reaching 85.98%. However, even in the case of free influenza vaccination in 2020 and 2021, the proportion of people over 70 years old is still <40%, which is far lower than that in developed countries (41). Further research is needed to find out what causes people who are willing to get vaccinated to fail to get vaccinated. This study used face-to-face and telephone interviews with community doctors or disease control personnel. With this survey method, the interviewees may be unwilling to express their true wishes directly in person or over the phone because they are worried about continuing to receive medical and health services. This may lead to an increase in the respondents' willingness to vaccinate in the survey results, rather than the real willingness to vaccinate. Studies have shown that appropriate monetary incentives can increase influenza vaccination rates (42).Whether some incentives can be considered to improve vaccination coverage in the elderly population in the Hangzhou region requires further discussion. The main reason for refusing influenza vaccinations in this study was “worry about AEFI after inoculation”. At present, the evidence on the complications of influenza vaccinations in the elderly in China is of poor quality, insufficient or outdated, and there is a lack of specific guidance documents or expert opinions on influenza vaccinations for people aged 65 and above (43). There is also a lack of similar related research in the Hangzhou area, especially for people with chronic diseases, and this paper fails to conduct further research.

## 5. Conclusion

The proportion of elderly people aged 70 and above who are willing to receive free influenza vaccine is higher in Hangzhou. But the level of knowledge about influenza, vaccine and trust in society is not high. Lack of knowledge about influenza and influenza vaccine is the most important reason for people aged 70 and above to refuse vaccination. The government should continue to improve the elderly's awareness and trust in society through medical staff, family members, television and radio media, and guide the elderly to actively vaccinate against influenza. Effective publicity should be carried out through the above channels to guide the elderly to actively vaccinate against influenza.

## Data availability statement

The original contributions presented in the study are included in the article/supplementary material, further inquiries can be directed to the corresponding author.

## Ethics statement

The studies involving human participants were reviewed and approved by Hangzhou CDC Institutional Review Board. The patients/participants provided their written informed consent to participate in this study.

## Author contributions

Conceived and designed the study: YL, XC, and WG. Obtained and organized the data: JuW, XC, and YX. Analyzed the data: FW and WJ. Contributed reagents, materials, and analysis tools: JD, XiZ, XuZ, and JiW. Wrote the manuscript: XC and YL. All authors contributed to the article and approved the submitted version.
